# Relationships between the home environment and physical activity and dietary patterns of preschool children: a cross-sectional study

**DOI:** 10.1186/1479-5868-5-31

**Published:** 2008-05-30

**Authors:** Nicola J Spurrier, Anthea A Magarey, Rebecca Golley, Fiona Curnow, Michael G Sawyer

**Affiliations:** 1 Department of Paediatrics and Child Health, Flinders University, Bedford Park, South Australia 5042, Australia; 2 Department of Nutrition and Dietetics, Flinders University, Bedford Park, South Australia 5042, Australia; 3 Research and Evaluation Unit, University of Adelaide, South Australia 5005, Australia

## Abstract

**Objective:**

To assess relationships between characteristics of the home environment and preschool children's physical activity and dietary patterns.

**Methods:**

Homes of 280 preschool children were visited and information obtained by direct observation and parent interview regarding physical and nutritional characteristics of the home environment. Children's physical activity, sedentary behaviour and dietary patterns were measured using standardised parent-report questionnaires. Associations were analysed using analysis of variance and correlation.

**Results:**

Parental physical activity (p = 0.03–0.008), size of backyard (p = 0.001) and amount of outdoor play equipment (p = 0.003) were associated with more outdoor play. Fewer rules about television viewing (p < 0.001) and presence of playstation (p = 0.02) were associated with more indoor sedentary time. Higher fruit and vegetable intake was associated with restricting children's access to fruit juice (p = 0.02) and restricting high fat/sugar snacks (p = 0.009). Lower intake of non-core foods was associated with restricting children's access to fruit juice (p = 0.007), cordial/carbonated drinks (p < 0.001) and high fat/sugar snacks (p = 0.003). Lower fruit and vegetable intake was associated with reminding child to 'eat up' (p = 0.007) and offering food rewards to eat main meal (p = 0.04). Higher intake of non-core foods was associated with giving food 'treats' (p = 0.03) and offering food rewards to eat main meal (p = 0.04). The availability of food groups in the home was associated with children's intake of these foods (fruit and vegetables, p < 0.001; fat in dairy, p = <0.001; sweetened beverages, p = 0.004–<0.001; non-core foods, p = 0.01–<0.001).

**Conclusion:**

Physical attributes of the home environment and parental behaviours are associated with preschool children's physical activity, sedentary behaviour and dietary patterns. Many of these variables are modifiable and could be targeted in childhood obesity prevention and management.

## Introduction

The prevalence of overweight and obesity in children have increased two and three-fold over the last decade in most developed countries [1-3]. Australian data suggest that one in five children are now above a healthy weight [3,4]. Population data from South Australia have shown that these rapid increases have also occurred in children as young as four years [5].

The childhood obesity epidemic appears secondary to changes in modern society resulting in increased availability of energy dense foods and reduced opportunities for physical activity [6,7]. There is growing evidence that many young children are consuming a diet inconsistent with recommendations and in addition, have inadequate levels of physical activity. For example, Australian dietary guidelines for children and adolescents recommend increasing the amount of fruit and vegetables consumed, reducing non-core snack foods, reducing consumption of sweetened beverages and choosing fat-reduced dairy from the age of 2 years [8]. Data from the Australian Food and Nutrition Monitoring Unit showed that children's total energy intake in a 24 hour period increased by 13% between 1985 and 1995, an increase largely consisting of non-core snack foods and sweetened drinks [9].

Australian physical activity guidelines recommend children spend a minimum of 60 minutes per day in moderate to vigorous physical activity and conversely spend a maximum of 120 minutes per day engaged in small screen entertainment [10]. A study of Australian 5–12 year olds showed that 15% of children this age did not fulfil the physical activity recommendations and 31% engaged in excessive electronic media use [11]. Consistent with this, a recent Australian study reported that 21% of 4 year olds spent greater than 2 hours engaged in TV viewing per day [12]. International studies report similar findings in preschool children. Reilly et al. used accelerometers to document physical activity of Scottish preschoolers. These young children spent only 20–25 minutes per day in moderate to vigorous physical activity and approximately 75% of the waking day engaged in sedentary activities [13]. Data from the US National Longitudinal Survey of Youth (1990–1998) showed that 41% of 2–3 year olds watched greater than 2 hours of television per day [14].

An ecological framework describes 'obesogenic' (or obesity promoting) environments in terms of macro and micro environments over which individuals have more or less control [15,16]. Characteristics of such environments are hypothesised to be direct determinants of children's physical activity and dietary patterns. For children, micro-environments include the family home, along with the school and local neighbourhood. Macro-environments could include transport, food supplies, the built environs, health care services and a wide range of government services and policies. Although many children spend time in care away from their home, especially for preschool children, the family home remains one of the principal environments, with family rules and preferences largely determining food availability and opportunities for physical activity [17].

Relationships between the environment and children's physical activity have been summarised in a recent systematic review [18]. Three of the 33 studies reviewed included preschool children; Baranowski et al., reported that preschool children were least active outdoors during the hottest months, Burdette et al., reported that mothers' perceptions of neighbourhood safety were not associated with time children spent playing outdoors and Sallis et al., reported that the more play spaces within walking distance, the higher the physical activity of the child [19-21]. Conversely, Trost et al., reported that parental support, active toys, television watching, park visitation and child competence were not significantly associated with time spent in active play during preschool sessions [22]. Whilst studies have consistently shown that parental role-modeling of physical activity is an important predictor of older children's physical activity, few studies have reported on this relationship in younger children [23,24].

Evidence supports positive relationships between greater numbers of televisions in the home and televisions in children's bedrooms, and greater amounts of time spent in sedentary activity [25]. A positive association has also been found between greater media use and children's body mass index (BMI), including a positive association between television viewing and preschool children's BMI [26,27]. More complex relationships around screen time have also been reported. For example, Salmon et al., reported that low physical activity was associated with enjoyment of Internet and computer use, a preference for watching television, parents who use computers/electronic games and having pay TV and electronic games available at home in 10–12 year olds [28]. Yalcin et al., showed a positive relationship between viewing habits of siblings and parents and their preschool children and also that leaving the household TV switched on when not being watched was associated with preschool children having greater viewing times [29].

While Birch and colleagues have comprehensively investigated the role of parental child-feeding practices on dietary intake and thus energy balance of very young children, other aspects of the family food environment have not been explored [30,31]. In a recent study of 5-to-6-year-old children, mother's who were less educated reported that the variety of fruits and vegetables available to them for purchase were limited and of poorer quality and that they were less likely to purchase fruit and vegetables because the family did not like them [32]. Hearn et al., assessed the presence of 10 varieties of fruit and 10 varieties of vegetables in the homes of 13 families who had school age children [33]. Weekend consumption of fruit and vegetables by children was associated with availability and access but weekday consumption was not.

In summary, previous research has predominantly focused on older children and most studies have assessed only one or two aspects of the home environment at any one time. The latter is problematic because conceptually it is possible that parents balance or compensate one aspect of the home environment with others. Assessing only one or two areas of the home environment does not give a comprehensive picture of what the home environment offers children. The aim of this study was to extend previous research conducted with older children by comprehensively describing characteristics of preschool children's home environments which may influence children's physical activity, sedentary behaviour and dietary patterns. Whilst we acknowledge the theoretical difficulty interpreting multiple comparisons, this approach gives a much broader and richer understanding of potential relationships between the home environment and children's physical activity and dietary patterns.

## Methods

### Subjects

Families were recruited through preschools in the Southern region of metropolitan Adelaide. In South Australia, Child and Youth Health (CYH) provide a free health screening program in the preschool setting which currently reaches approximately 85% of preschool children. Initial contact with parents was made by nurses undertaking this program in the region. Nurses advised parents about the study and if parents were willing to participate, nurses forwarded contact details to the researchers. Parents were recruited from a total of 49 preschools (out of a possible 55 preschools in the recruitment area). Of 516 eligible parents whose children were screened during the recruitment period, 331 parents (64%) provided a telephone number and could be contacted by the researchers. Of these 331 parents, 280 (85%) agreed to participate in the study and were visited at home by the research team, giving an overall response rate of 54%.

### Data Collection

Data were collected by five research interviewers who individually visited families in their homes after arranging a suitable time for the visit. All interviewers were trained by undertaking a site visit to a family not involved in the study to review the assessment procedure in detail. Interviewers were also provided with comprehensive written instructions to ensure a standardised procedure was followed. Characteristics of the home environment were assessed by direct observation and by parent interview. Visits took an average of 75 minutes. Informed consent was obtained from all participating parents and assent from all children. The study complied with the Declaration of Helsinki (1996) and was approved by the Flinders University Social and Behavioural Research Ethics Committee and the Children, Youth and Women's Health Service Research Ethics Committee. Data were collected between February and September 2005.

### Measures

*The Physical and Nutritional Home Environment Inventory*. A 74 item inventory was developed for this study (Additional file 1). Items were included which in previous studies were associated with children's physical activity levels, sedentary behaviour, dietary patterns or body mass index or had potential for modification [34]. Thirty-three items were hypothesised to be associated with either children's physical activity or sedentary behaviour (called physical home environment items) and 41 items were hypothesised to be associated with children's dietary patterns (called nutritional home environment items). Approximately half the items were assessed by direct observation, the rest by parental report.

#### Direct observation

Physical home environment items included size of back yard and lawn area (measured to the nearest m^2 ^using a circular metered ruler), the number of pieces of outdoor play equipment, presence of a paved area for bike riding, number and positioning of televisions and presence of other types of small screen entertainment. Nutritional home environment characteristics included quantity of fruit and vegetables, high fat/sugar non-core snack foods, fat content of dairy products and sweetened drinks present in kitchen pantries, refrigerators and freezers. These food groups relate to four key messages for children's nutrition from the Australian Guide to Healthy Eating (AGHE); increase fruit and vegetable consumption, low fat rather than full fat dairy products for children older than two years, reduce intake of non-core snack foods and consume water or milk as main beverage [35]. Frozen, dried and canned, fruits and vegetables were included and there was a three kilogram upper-limit for potatoes. Onion, garlic, chilli, capers, limes and lemons were not included as these were considered flavourings. Detailed instructions regarding what constituted high-fat sugared and salted snacks were available for home interviewers. Individual food groups were weighed on standardised kitchen scales and these amounts were recorded on 4-point Likert scales (e.g., all fresh, frozen and canned vegetables were weighed and added together). Scale categories were based on quantities of food groups required by an average size family in a week as recommended by the AGHE (see Additional file 1 for details).

#### Parent report

Other items in the inventory required parental report and details regarding what was meant by each item and individual Likert scales are fully described in Additional file 1. In brief, physical home environment characteristics included family use of active transport, parental role-modelling, presence of community facilities in close proximity (library, play-ground), extra-curricular activities for preschool children (swimming, dance classes, sport classes etc.), family rules about use of television and use of labour saving devices. Nutritional home environment characteristics included parental food providing behaviours; for example, size of the average meal (parents were shown the size of the average dinner plate divided into quarters), numbers of snacks per day, using food as reward, encouraging children to 'eat up' and restricting children's access to different food groups.

##### The Outdoor Playtime and Small Screen Entertainment Checklist

This brief twelve item questionnaire was based on the Outdoor Playtime Checklist developed by Burdette et al. [36]. The latter is a six item checklist measuring time spent by preschool children in outdoor playtime around the home and in other outdoor areas and has been validated against accelerometer data [36]. Parents respond on a 5-point Likert scale (0 minute, 1–15 minutes, 16–30 minutes, 31–60 minutes and 60 + minutes, scored as 0 to 4 respectively) for three time periods (wake-up time until noon, noon until 6 pm, 6 pm until bedtime). Item scores are summed to give a continuous variable (0 to 24) with high scores indicating more outdoor playtime. We added six items focusing on sedentary activity to the original questionnaire. The new items were, 'How much time did your child spend watching television (including videos and DVDs)?' and 'How much time did your child spend playing Play-Station/X-Box/computer games (including watching a friend/brother/sister play)?' The three time periods used for outdoor playtime were used for both items (making six items in total) with parents responding on a 5-point Likert scale (0 minutes, 1–60 minutes, 61–120 minutes, 121–180 minutes and 180+ minutes, scored 0 to 4 respectively). A different time scale was used because this both reflected the National Physical Activity Guidelines recommendation of less than 2 hours of television viewing per day and the fact that children of this age have been shown to watch approximately 2–3 hours of television per day; Yalcin et al. reported preschool children watched 2.19 ± 1.84 hours per day and Burdette and Whitaker reported 190 ± 128 minutes per day [10,29,37]. Item scores are summed giving a continuous variable (0 to 24) with high scores representing more time spent in small screen entertainment.

##### The Children's Dietary Questionnaire

This 24 item parent report questionnaire (available from the authors) assesses children's dietary patterns in relation to key elements of the AGHE known to be areas for improvement based on child dietary intake data from the 1995 National Nutrition Survey [38,39]. This approach was chosen over a standard food frequency questionnaire because the Children's Dietary Questionnaire generates four continuous subscales which relate directly to current dietary recommendations. Responses are made on five and six point Likert scales generating four food group scores; *fruit and vegetable *score (high score representing greater variety and amount of fruit and vegetables), *fat from dairy products *score (high score representing greater intake of full fat dairy products), *sweetened beverages *score (high score representing greater intake of fruit juice, cordial and soft drink) and *non-core foods *score (high score representing greater intake of high fat/high sugar snack foods). Scores do not represent actual number of portions of different food groups but are continuous scales reflecting dietary patterns. In terms of psychometric properties, all subscales of the Children's Dietary Questionnaire have demonstrated satisfactory test-retest reliability (intraclass correlation coefficient 0.51 to 0.90) and an ability to detect change in the expected direction following a weight management intervention. The *fruit and vegetable *and *non-core foods *subscales have shown good internal consistency (alpha 0.76 and 0.62 respectively) and item:total correlations greater than 0.2, whilst the *fat from dairy products *and the *sweetened beverages *subscales performed poorly in this respect (likely due to the small number of items in these subscales). Criterion validity suggests the Children's Dietary Questionnaire has the ability to distinguish positive and negative dietary risk at the group level but not the individual level.

##### Sociodemographic and anthropometric data

A brief sociodemographic questionnaire was completed by all parents. Children's weight to the nearest 0.1 of a kilogram was measured in light clothing (no shoes) using Propert Model 1650 electronic scales and children's height was measured in stocking feet using a Cottman SBA portable stadiometer. Body mass index was calculated as weight in kilograms divided by height in metres squared (weight (kg)/(height (m))^2^) and weight status (healthy weight, overweight and obese) determined using age and gender specific International Obesity Task Force cut-points [40].

### Statistical Analysis

Data were analysed using STATA version 8, statistical software [41]. Subscale scores for outcome variables (physical activity patterns and dietary patterns) were assessed for normality, and means, standard deviation and range were calculated. All subscale scores for the Outdoor Playtime and Small Screen Entertainment Checklist and the CDQ were normally distributed except the sweetened beverages subscale. A square root transformation was made to the later, prior to analyses. Analysis of variance (ANOVA) was used to assess relationships between the majority of the 33 physical home environment characteristics and subscales of the Outdoor Playtime and Small Screen Entertainment Checklist and between the 41 nutritional home environment characteristics and the four subscales of the Children's Dietary Questionnaire. Post hoc tests using the Bonferroni procedure were employed to identify the statistical significance of differences between pairs of categories. Pearson correlation was used if the explanatory variable was continuous. Because theoretically items in the Physical and Nutritional Home Inventory were not necessarily related, individual items were analysed as separate explanatory variables. Because of the large number of analyses undertaken, descriptive statistics and p values are provided in the tables only for the statistically significant associations (p < 0.05). Although there is a theoretical risk of a type I error when presenting multiple comparisons, as recommended by Rothman we have not undertaken statistical corrections for the ANOVA results presented (which increase the risk of a type II error) but have provided full information on the number of analyses conducted by including all items for the Physical and Nutritional Home Inventory in Additional file 1[42]. Where particular patterns could be determined amongst the associations found to be statistically significant, items were grouped under descriptive headings in the tables.

## Results

### Sample characteristics

Children were all less than five and a half years of age (mean = 4.8 years ± 0.21, range = 4.1 – 5.4 years) with boys and girls equally represented. Most children lived in two parent households, with only 10% living in single parent households, significantly less than the state average of 18% [43]. Similarly 18% of children live in low income families (< $20,000/year) in metropolitan Adelaide whilst only 8% were in this category in our sample [43]. The proportion of children identified as overweight and obese (15% and 6% respectively) was consistent with population surveys [4,5].

### Physical home environment characteristics and children's Outdoor Playtime and Small Screen Entertainment Checklist scores (Tables 1 and 2)

**Table 1:** Relationships between the physical home environment and children's outdoor playtime for the previous day (n = 276) (ANOVA used for categorical variables and Pearson correlation for continuous variables).

Descriptive Construct	Physical Home Environment Item	Children's Physical Activity Mean *outdoor playtime *score (range = 0–18)				p value
		≥ Once a week	Fortnightly to monthly	Couple of times a year	Nil in last 12 months	
Role modelling	Mother's frequency of walking >30 mins per day	8.8^a^	7.3^ab^	8.2^ab^	6.0^b^	0.008
	Mother's frequency of organised sport	9.0^a^	8.9^ab^	8.5^ab^	7.5^b^	0.04
	Father's frequency of walking >30 mins per day	8.7^ab^	7.8^ab^	9.5^a^	7.2^b^	0.03
						
		Very much	Quite a bit	Not very much	Not at all	
Physical attributes of the home	Presence of labour saving devices	8.3^ab^	8.1^ab^	9.1^a^	7.1^b^	0.04
	Size of backyard	291 ± 358.3 (0–3000) m2 [r = 0.20^c^]				0.001
	Number of items of outdoor play equipment	11 ± 3.15 (2–19) items of equipment [r = 0.17^c^]				0.003

^a,b^Superscripts indicate which categories show a statistically significant difference using Bonferroni correction: same letter indicates no difference, different letter indicates a difference.

^c^Pearson correlation coefficient

**Table 2:** Relationships between physical home environment and children's amount of small screen entertainment for the previous day using ANOVA (n = 280).

Descriptive Construct	Physical Home Environment Item	Children's Sedentary Activity Mean *small screen entertainment *score (range = 0–11)				p value
		Frequently	Sometimes	Occasionally	Rarely/Never	
Physical activity (transport/play/sport outside home)	Frequency child attends swimming lessons	2.7^a^	2.6^ab^	3.6^ab^	3.6^b^	0.002
Rules about small screen entertainment	Frequency that TV is left on in home	3.8^a^	3.3^ab^	2.9^b^	2.5^b^	<0.001
	Parents set rules about TV viewing	2.5^a^	3.6^b^	3.6^b^	4.1^b^	<0.001
	Parents limit exposure to TV advertising	2.6^a^	2.7^a^	3.7^b^	3.7^b^	<0.001
						
			Yes	No		
Presence of small screen entertainment in home	Play station in home		3.5	2.9		0.02
	Internet connection in home		3.0	3.7		0.01
	Computer in home		3.1	3.9		0.07

^a,b ^Superscripts indicate which categories show a statistically significant difference using Bonferroni correction: same letter indicates no difference, different letter indicates a difference.

Higher *outdoor playtime *scores were significantly associated with greater frequency of mothers' walking and mothers' involvement in organised sport. The direction of the relationship between father's frequency of walking and outdoor playtime was less easily interpretable. Higher *outdoor playtime *scores were significantly associated with greater backyard size and more items of outdoor play equipment in the backyard although the size of the correlations were relatively small (0.17 and 0.20 respectively). In contrast, parental role-modelling of physical activity and characteristics of the backyard were not associated with children's *small screen entertainment *scores.

Whilst more family rules about TV viewing were associated with lower *small screen entertainment *scores, the actual number of TV's present in the household was not. Whilst the presence of a 'play-station' available for children was significantly associated with higher *small screen entertainment *scores, presence of an internet connection in the home was significantly associated with lower *small screen entertainment *scores. None of the small screen entertainment items in the physical home environment inventory were associated with children's *outdoor playtime *scores.

### Nutritional home environment characteristics and Children's Dietary Questionnaire scores (Tables 3, 4, 5, 6)

**Table 3:** Relationships between the nutritional home environment and children's intake of fruit and vegetables using ANOVA (n = 279).

Descriptive Construct	Nutritional Home Environment Item	Children's Dietary Patterns Mean *Fruit and Vegetable *score (range = 0–21)				p value
		≤ 1/4 plate	1/3 plate	1/2 plate	> 1/2 plate	
Parental behaviours associated with food	Average portion size served to child	8.3^a^	10.6^b^	10.9^b^	11.0^b^	0.002
						
		Frequently	Sometimes	Occasionally	Rarely/Never	
	Food allowed to be eaten in front of TV	9.3^a^	10.1^ab^	10.4^ab^	11.8^b^	0.01
	Acceptance of wasted food	8.8^a^	9.9^a^	10.2^a^	11.8^a^	0.04
	Remind child to 'eat up'	9.7^a^	11.6^b^	10.5^ab^	12.3^b^	0.007
	Offer food rewards/incentives to eat main meals	9.0^a^	10.9^a^	10.9^a^	10.5^a^	0.04
	Restrict fruit juice	10.9^a^	10.4^ab^	11.3^a^	9.2^b^	0.02
	Restrict high fat/sugar snack foods	11.1^a^	9.4^b^	9.4^ab^	8.4^ab^	0.009
	Restrict second helpings	14.4^a^	9.6^a^	11.9^a^	10.1^a^	0.04
Availability of food in home	Take-away food purchased	8.5^a^	9.8^a^	10.8^a^	11.3^a^	0.03
						
		0–2.9 kg	3–5.9 kg	6–7.9 kg	≥8 kg	
	Amount of fruit	9.2^a^	11.0^b^	11.7^b^	11.9^b^	<0.001
		0–1.9 l	2–2.9 l	3–3.9 l	≥4 l	
	Amount of fruit juice in home	10.4^ab^	11.6^a^	9.2^ab^	8.9^b^	0.01
		0 boxes	0.5 boxes	1–2 boxes	>2 boxes	
	Amount of muesli bars/breakfast bars in home	10.9^a^	10.9^ab^	9.2^b^	10.5^ab^	0.04

^a,b ^Superscripts indicate which categories show a statistically significant difference using Bonferroni correction: same letter indicates no difference, different letter indicates a difference.

**Table 4:** Relationships between the nutritional home environment and children's intake of fat from dairy products (n = 276) (ANOVA used for categorical variables and Pearson correlation for continuous variables).

Descriptive Construct	Nutritional Home Environment Item	Children's Dietary Patterns Mean *Fat in Dairy Products *score (range = 3–20)				p value
		Frequently	Sometimes	Occasionally	Rarely/Never	
Parental behaviours associated with food	Reward good behaviour with food	4.8^a^	4.0^a^	3.1^a^	3.7^a^	0.04
	Restrict second helpings	1.3^a^	3.2^a^	2.9^a^	3.9^a^	0.04
						
		0–1.9 l	2–2.9 l	3–3.9 l	≥4 l	
Availability of food in home	Amount of fruit juice in home	3.5^a^	4.6^b^	3.2^ab^	3.5^ab^	0.02
		Full fat	Reduced fat	Mixed (reduced fat/low fat)	Low fat	
	Type of dairy in home	4.1^a^	1.9^b^	3.8^a^	2.3^ab^	<0.001
	Number of snacks per day	2.9 ± 1.15 (0–8) snacks per day [r = 0.16^c^]				0.008

^a,b ^Superscripts indicate which categories show a statistically significant difference using Bonferroni correction: same letter indicates no difference, different letter indicates a difference.

^c ^Pearson Correlation coefficient.

**Table 5:** Relationships between the nutritional home environment and children's intake of sweetened beverages using ANOVA (n = 280).

Descriptive Construct	Nutritional Home Environment Item	Children's Dietary Patterns Mean square-root of *Sweetened Beverages *score (range = 0–6.9)				p value
		Frequently	Sometimes	Occasionally	Rarely/Never	
Parental behaviours associated with food	One or both parents eat main meal with children	2.8^ab^	2.5^a^	3.2^ab^	3.4^b^	0.05
	Evening meal in front of TV	3.6^a^	2.8^ab^	2.8^b^	3.3^ab^	0.02
	Reward good behaviour with food	4.2^a^	2.9^b^	3.4^ab^	3.2^ab^	0.02
	Restrict fruit juice	2.6^a^	3.0^ac^	3.9^b^	3.7^bc^	<0.001
	Restrict carbonated drink/cordial	3.1^a^	3.6^a^	3.6^a^	2.9^a^	0.05
						
		0–1.9 l	2–2.9 l	3–3.9 l	≥4 l	
Availability of food in home	Amount of fruit juice in home	2.9^a^	3.6^b^	4.2^b^	3.5^ab^	<0.001
		0–1.9 l	2–4.9 l	5–9.9 l	≥10 l	
	Amount of cordial and carbonated drink in home	2.8^a^	3.7^b^	3.2^ab^	3.4^ab^	0.004

^a,b,c ^Superscripts indicate which categories show a statistically significant difference using Bonferroni correction: same letter indicates no difference, different letter indicates a difference.

**Table 6:** Relationships between the nutritional home environment and children's intake of non-core foods (n = 272) (ANOVA used for categorical variables and Pearson correlation for continuous variables).

Descriptive Construct	Nutritional Home Environment Item	Children's Dietary Patterns Mean *Non-core Foods *score (range = 2–43)				p value
		Frequently	Sometimes	Occasionally	Rarely/Never	
Parental behaviours associated with food	Give child food 'treats'	19.7^a^	21.4^a^	19.0^a^	17.1^a^	0.03
	Other carers give child food 'treats'	19.1^a^	23.4^b^	19.5^a^	18.1^a^	0.001
	Offer food rewards/incentives to eat main meals	19.4^ab^	22.1^a^	19.7^ab^	18.6^b^	0.04
	Restrict fruit juice	17.7^a^	21.9^b^	19.3^ab^	20.8^b^	0.007
	Restrict high fat/sugar snack foods	18.3^a^	21.5^b^	22.7^b^	20.2^ab^	0.003
	Restrict carbonated drink/cordial	18.3^a^	23.4^b^	21.5^ab^	18.5^a^	<0.001
	Snacks allowed to be eaten in front of TV	22.0^a^	19.9^ab^	18.2^b^	17.7^b^	0.006
	Other meals allowed to be eaten in front of TV	21.1^a^	19.7^ab^	22.1^a^	17.5^b^	0.002
	Child helps prepare food	18.1^a^	19.7^a^	18.8^ab^	23.5^b^	0.004
Availability of food in home	Take-away food purchased	21.5^a^	20.5^a^	20.2^a^	15.5^b^	0.005
						
		0–99 grams	100–299 grams	300–699 grams	≥700 grams	
	Amount of chips, snack savoury biscuits, salted nuts in home	17.5^a^	19.1^ab^	19.8^ab^	21.7^b^	0.01
		0–119 grams	120–399 grams	400–799 grams	≥800 grams	
	Amount of lollies, sweets, chocolates in home	18.0^a^	18.6^ac^	22.3^bc^	22.8^b^	<0.001
		0 boxes	0.5 boxes	1–2 boxes	>2 boxes	
	Amount of muesli bars/breakfast bars in home	17.4^a^	19.7^ab^	21.6^b^	21.6^b^	0.001
		0–139 grams	140–499 grams	500–999 grams	≥1000 grams	
	Amount of cake/biscuits in home	17.2^a^	18.9^ac^	21.6^bc^	23.3^b^	<0.001
	Number of snacks per day	2.9 ± 1.15 (0–8) snacks per day [r = 0.23^d^]				<0.001

^a,b,c ^Superscripts indicate which categories show a statistically significant difference using Bonferroni correction: same letter indicates no difference, different letter indicates a difference.

^d ^Pearson correlation coefficient

Higher *fruit and vegetable *scores were associated with the following parental behaviours; larger overall size of meal-time serve, less acceptance of wasted food, less reminders to 'eat up', less use of food rewards and incentives, not allowing child to eat in front of TV and more frequent restriction of extra foods (fruit juice, high fat/sugar snacks and second helpings). There was a strong positive association between the amount of fruit and vegetables available in the family home and higher *fruit and vegetable *scores. Conversely, greater quantities of fruit juice and muesli bars/breakfast bars kept in the home were associated with lower *fruit and vegetable *scores.

Having full fat dairy products available in the home was associated with a higher *fat in dairy products *score. Higher *fat in dairy products *score was also associated with greater number of snacks children consumed in a day. Although a statistically significant association between availability of fruit juice in the home and the *fat in dairy products *score was found, the mean scores did not give a clear indication of the direction of the association. In terms of parental food-related behaviours, more frequent food rewards for good behaviour was associated with higher *fat in dairy products *scores as was less restriction of second helpings.

Parental behaviours which were significantly associated with greater intake of *sweetened beverages *were; less frequent family meals (child and parent eats together), evening meal eaten in front of TV, and more frequent use of food rewards for good behaviour. Conversely greater restriction by parents of fruit juice, carbonated drinks and cordial was associated with lower *sweetened beverages *scores. There was a strong positive association between having greater amounts of fruit juice, carbonated drinks and cordial in the home and children's intake of these beverages.

More frequent use of food 'treats' by parents and other carers and using food as a reward to eat main meal were associated with higher *non-core food *scores. Conversely, parental restriction of sweetened beverages (including fruit juice) and high fat/sugar snack foods was associated with lower *non-core food *scores. Eating meals and snacks in front of the TV was associated with higher *non-core foods *scores. Higher *non-core foods *scores were associated with more frequent purchase of take-away food and keeping more of these non-core foods in the family pantry.

## Discussion

This study aimed to assess relationships between home environment characteristics and physical activity and dietary patterns of preschool children. Outdoor playtime, used as a proxy for physical activity, was positively associated with greater frequency of parental participation in physical activity, particularly mother's participation. The importance of parental modelling of physical activity has been demonstrated in other studies. Ferreira at al. in a review of correlates of children's physical activity, summarised 29 studies investigating parental physical activity and 29 and 31 studies investigating separately father's and mother's physical activity respectively [44]. In contrast to our study, Ferreira et al. concluded that father's level of physical activity had a stronger relationship with children' physical activity than mother's. This may have been because in this review, outcomes of studies of children aged 3–12 were combined. Mother's physical activity may be a stronger influence on the physical activity of younger children when children are of an age when mothers are more likely than fathers to spend time at home. Similarly, at this age it is plausible that having a parent accompany a child undertake outside activities is more important than children actually modelling parental behaviour. Regardless of the mechanism, encouraging parental physical activity appears to be an important and practical way of increasing both the physical activity of preschoolers and older children.

Children's physical activity was also positively associated with more outdoor play equipment and a larger backyard size. Whilst the size of these correlations were small, these findings are consistent with a number of other studies [44,45]. A more interesting outdoor environment may encourage children to spend time in active outdoor play, but it is equally plausible that parents of sedentary children respond by investing less in such activities. Similarly, a larger backyard size may increase time children spend outdoors, but it is also plausible that more active families purchase homes with larger outdoor areas. In contrast, there was no association between presence of a children' playground close to the home and children's outdoor play, suggesting that the home rather than the neighbourhood environment is more important at this age. There was no significant association between amount of time spent in organised physical activities such as swimming lessons, dance, kindergym or gymnastics and children's outdoor playtime. These relatively costly activities generally take up only small amounts of time in the daily lives of preschool children (in our study 39% and 29% of children attended a swimming lesson or another form of organised physical activity once a week) and do not appear to influence the time children spend in free play at home. Finally, our inventory included an item about dog ownership. Dog ownership was not associated with outdoor playtime of preschoolers in our study, consistent with one other study, which showed that whilst dog ownership was associated with physical activity of older children, it was not associated with walking or cycling of 5–6 year old children [46].

Consistent associations were found between less screen time and greater parental monitoring of television use in the home and more rules about television viewing. In contrast to previous research, the number of televisions present in the family home was not associated with screen time [26,29]. One plausible explanation is that the effect of this variable is much weaker now the majority of homes have multiple televisions (47% of families in our sample had three or more televisions compared to only 10% of families of preschoolers in the study of Yalcin et al. conducted in 1999) [29]. We also did not find an association between the presence of television in preschool children's bedrooms and screen time. Yalcin et al. also reported this negative finding [29]. This contrasts to the literature in older children where associations have been found between presence of television in children's bedrooms and both sedentary behaviour and obesity [12,47]. Perhaps having a television in the bedroom is not such an important influence on younger children's viewing patterns compared with older children because younger children do not spend as much time involved in independent activities in their bedrooms. Another unexpected finding was that the presence of an Internet connection was significantly associated with lower levels of small screen entertainment time and a similar but non-significant association was found for presence of a computer in the home (p = 0.07). One plausible explanation for this finding is that preschool children do not use computer or the Internet for entertainment. Alternatively, we did not control for socio-economic status, and children of more affluent families may both have less screen time and be more likely to have computer and Internet available.

Previous research suggests that physical activity and sedentary behaviour represent different constructs [48]. Our study demonstrated that a different set of explanatory variables were associated with the amount of outdoor playtime of preschool children compared to time spent engaged in small screen entertainment consistent with this premise.

The most consistent nutritional variables associated with children's dietary patterns were the types and amounts of foods located within the family home. Other than the small study of Hearn et al., which focused solely on fruit and vegetable intake, this is the first time an association between children's intake and food availability in the home has been documented [33]. The association was found for both intake of core food groups (fruit and vegetables) and non-core food groups (sweet and salty snacks and sweetened beverages). A plentiful supply of fruit, vegetables and low fat dairy foods along with reducing the presence of fruit juice, sweetened beverages and non-core foods, were associated with higher intake of fruit and vegetables and lower intake of foods with less nutritional value. From a practical perspective, counselling parents regarding the types and amounts of foods stocked in the home may be more effective and less stigmatising than focusing on dietary patterns or body weights of individual children for either managing a child who is overweight or undertaking preventive education with parents.

Consistent with previous research, our study found significant associations between a variety of parental behaviours around food provision and children's dietary patterns [49]. Higher intake of fruit and vegetables was associated with parental behaviours restricting access to less healthy foods (fruit juice, high fat/sugar snacks) and restricting second helpings. In comparison, lower intakes of fruit and vegetables were associated with greater use of coercive parental behaviours such as offering food rewards to eat main meal and reminding child to 'eat up'. The causal direction of the relationship cannot be determined from this cross-sectional data. One plausible explanation is that parents of children who were poor consumers of fruit and vegetable from a younger age have responded by using these coercive techniques. Alternatively, Birch et al. argue from their experimental data that coercive behaviours can lead children to favour less healthy foods and specifically that restricting children's intake of less healthy foods can unintentionally lead to a preference for these foods [50,51]. Our data suggests that coercive and restrictive parental behaviours should be considered separately. For example, we also showed that greater use of restrictive behaviours by parents was associated with lower intake of less healthy foods (sweetened beverages and non-core foods). Conversely, the use of food treats either by parent or other carers (a coercive behaviour), was associated with higher intake of less healthy foods. Thus parental restriction of access by children to less healthy foods appears to be associated with better dietary patterns whilst coercive behaviours are associated with poorer dietary patterns.

Finally, the positive relationships between allowing children to eat in front of the television and greater intake of less healthy food groups (sweetened beverages and non-core foods) and lower intake of more healthy food groups (fruit and vegetables) deserves mention. Post hoc analyses showed a small but significant positive correlation between the *non-core food *score and the *small screen entertainment *score (r = 0.14 and p = 0.02) and a small but significant negative Pearson correlation between the *fruit and vegetable *score and the *small screen entertainment *score (r = -0.17, p = 0.004). These findings suggest the relationship between television viewing and the development of obesity may well be partially mediated by dietary patterns in addition to reduced energy expenditure. This is supported by a recent randomised controlled trial of reducing television viewing to treat overweight 4–7 year olds where change in television viewing was related to the change in energy intake and not to changes in physical activity [52].

The study has a number of limitations. The data is cross-sectional and the temporal direction of associations cannot be determined. All analyses were univariable and did not take into account potential confounding variables such as socioeconomic status. The sample size was relatively small and parents were of higher socioeconomic status compared to the South Australian average. Whilst studies generally report an association between more healthy dietary patterns and higher socioeconomic status, recent studies fail to confirm an association between socioeconomic status and children's physical activity [32,53].

The home environment inventory was analysed in terms of individual items rather than subscale scores and responses to individual items are inherently less stable than summed scores. This approach was taken because conceptually there was no theoretical basis on which to assume some items were related and could be summed. For example, just because parents may score high on one item (e.g. presence of non-core snack foods in the home) does not necessarily mean that they would score high on another item (e.g., presence of sweetened beverages in the home). The theoretical objection to presenting multiple comparisons (i.e., that a number of significant findings would be expected by chance) has been addressed by providing the total number of items (i.e., comparisons) in Additional file 1[42].

The relative newness of this area of research meant that we had to develop a questionnaire to measure the home environment, use a recently developed questionnaire to measure preschool children's dietary patterns and add supplementary questions to the physical activity questionnaire in order to measure sedentary activity. In addition, assessing the inter-rater reliability of the Physical and Nutritional Home Environment Inventory was beyond the scope of this study. Given the limited information regarding the validity of the questionnaires used, the results of this study need careful interpretation and ideally, replication.

The study has a number of strengths. This is one of few studies to measure elements of the home environment by direct observation. Other studies have relied on parental report regarding numbers of televisions, availability of outdoor play equipment or household food [19,23,28,32]. In addition, the study included both assessments of dietary and physical activity patterns rather than focusing on only one side of the energy balance equation. Practical aspects of ensuring a healthy lifestyle for children by parents were examined and this has resulted in data that has real clinical utility for health professionals dealing with overweight children and their families. This is particularly true for the consumption of fruit and vegetables and non-core foods where our study found many significant associations with children's intake. Addressing these factors is particularly important because it is generally the over-consumption of non-core foods which result in excessive energy intake in children [54].

## Conclusion

The key finding of this study was that many practical and potentially modifiable aspects of the home environment were associated with preschool children's physical activity and dietary patterns. Parents are important in terms of role-modelling physical activity, providing a safe and interesting backyard for children to play in, setting rules about how small screen entertainment is used in the home, behavioural approaches to family food consumption, and providing healthy food choices in the home. Preschool years are a key time as children spend a large proportion of time at home and this study supports focusing on the family home environment for obesity prevention and management.

## Competing interests

The authors declare that they have no competing interests.

## Authors' contributions

NJS, AAM, RG and MGS contributed to the conception and design of the study. FC and RG contributed to data acquisition. NJS and AAM undertook data analysis and interpretation. NJS wrote the original draft and incorporated subsequent comments from all other authors. All authors have read and approved the final manuscript.

## Supplementary Material

Additional file 1 Items included in the Physical and Nutritional Home Environment Inventory. This file includes all the items in the Physical and Nutritional Home Environment Inventory and the response options and Likert scales for each item. Click here for file
