# Supplementary material for: Relationships between the home environment and physical activity and dietary patterns of preschool children: a cross-sectional study
Source: Int J Behav Nutr Phys Act. 2008 May 30;5:31. doi: 10.1186/1479-5868-5-31 (PMC2432071; doi:10.1186/1479-5868-5-31)
Supplement: Additional file 1 — Items included in the Physical and Nutritional Home Environment Inventory. This file includes all the items in the Physical and Nutritional Home Environment Inventory and the response options and Likert scales for each item. [file 1479-5868-5-31-S1.doc]

Items included in the Physical and Nutritional Home Environment Inventory

| Physical home environment item | Likert scale | | | |
| --- | --- | --- | --- | --- |
|  |  |  |  |  |
| 1. How often does your child travel to childcare/preschool by walking, riding bicycle/tricycle, public transport? (bus/train/tram) | frequently | sometimes | occasionally | rarely/never |
| 1. How often does your child travel to childcare/preschool by car, riding in a pram, parent’s bike seat? | frequently | sometimes | occasionally | rarely/never |
| 1. How often does the child’s mother (mother-figure) go for a walk of more than 30 minutes? | >1x/week | 1x/fortnight  - 1x/month | couple of times/year | nil in last 12 months |
| 1. How often does the child’s mother (mother-figure) play sport/go running/go swimming/attend a gym/go cycling? | >1x/week | 1x/fortnight  - 1x/month | couple of times/year | nil in last 12 months |
| 1. How often does the child’s father (father-figure) go for a walk of more than 30 minutes? | >1x/week | 1x/fortnight  - 1x/month | couple of times/year | nil in last 12 months |
| 1. How often does the child’s father (father-figure) play sport/go running/go swimming/attend a gym/go cycling? | >1x/week | 1x/fortnight  - 1x/month | couple of times/year | nil in last 12 months |
| 1. How often does your child walk with you to do an errand (eg. to the local shops, to post a letter)? | >1x/week | 1x/fortnight  - 1x/month | couple of times/year | nil in last 12 months |
| 1. Is there a children’s playground within 10-minutes walking distance of your house? | yes | no |  |  |
| 1. On average, how many times per week is your child taken to any playground outside of the home? | ≥ 4x | 2-3x | once/week | < once/  week |
| 1. Do you own a dog? | yes | no |  |  |
| 1. How often does your child attend swimming lessons? | frequently | sometimes | occasionally | rarely/never |
| 1. How often does your child attend any type of dance, kindergym, gymnastics, or any other organised physical activity? | frequently | sometimes | occasionally | rarely/never |
| 1. How often does your family use the local book and/or toy library? | frequently | sometimes | occasionally | rarely/never |
| 1. How many televisions are in your household? | continuous variable | | | |
| 1. Are there any televisions in view of where main meals are eaten? | yes | no |  |  |
| 1. Is there a television in the parents’ bedroom? | yes | no |  |  |
| 1. Is there a television in your preschool child’s bedroom? | yes | no |  |  |
| 1. Is there a television in your other children’s bedroom/s? | yes | no |  |  |
| 1. Does your family have cable/satellite TV? | yes | no |  |  |
| 1. Does your family own a video-player (VCR)? | yes | no |  |  |
| 1. Does your family own a DVD player? | yes | no |  |  |
| 1. Does your family have a computer at home? | yes | no |  |  |
| 1. Does your household have an internet connection? | yes | no |  |  |
| 1. Does your preschool child have access to a play-station/X-box at home? | yes | no |  |  |
| 1. How often is your television left on, whether or not it is being watched? | frequently | sometimes | occasionally | rarely/never |
| 1. How often do the adults in your family watch the television news during the evening meal? | frequently | sometimes | occasionally | rarely/never |
| 1. How much do the adults in your household set rules about children’s television viewing? | very much | quite a bit | not very much | not at all |
| 1. How much do you try to limit your preschool child’s exposure to television advertising? | very much | quite a bit | not very much | not at all |
| 1. How much does your family use labour saving devices (such as dishwashing machine, remote control gate, electric roller door, leaf blower etc)? | very much | quite a bit | not very much | not at all |
| 1. What is the size of the yard area available for play by the child (m²)? | continuous variable | | | |
| 1. What is the size of the lawn area (m²)? | continuous variable | | | |
| 1. Does the yard have a paved area that your child can use for bike riding? | yes | no |  |  |
| 1. What other sporting/play equipment do you have available for your preschool child? (The complete list is available from the authors) | continuous variable | | | |

| Nutritional home environment items | Likert scale | | | |
| --- | --- | --- | --- | --- |
|  |  |  |  |  |
| 1. What is the average time that you spend preparing the evening meal? | 0-15 min | 15-30 min | 30-60 min | >60 min |
| 1. How many times a week does one or both parents eat the main meal of the day with your child/children? | 1-2x | 3-4x | 5-6x | ≥7x |
| 1. How many meals does your child eat per day? | continuous variable | | | |
| 1. How many snacks does your child eat per day? (anything eaten outside of the main meal) | continuous variable | | | |
| 1. How often does your child eat the evening meal in front of the television (turned on)? | frequently | sometimes | occasionally | rarely/never |
| 1. How often does your child eat other meals in front of the television (turned on)? | frequently | sometimes | occasionally | rarely/never |
| 1. How often does your child eat snacks in front of the television (turned on)? | frequently | sometimes | occasionally | rarely/never |
| 1. What is the average portion size served out for your child for their evening meal? | ≤ 1/4 average dinner plate | 1/3 average dinner plate | 1/2 average dinner plate | > 1/2 average dinner plate |
| 1. How often is food consumed directly out of the packet/pot/fridge by family members? | frequently | sometimes | occasionally | rarely/never |
| 1. How often does your family purchase takeaway food, which your child also eats? | frequently | sometimes | occasionally | rarely/never |
| 1. How much does your family accept wasted food? | very accepting | accepting | unhappy about waste | very unhappy |
| 1. How often during an average meal do you remind your child to ‘eat up’? | frequently | sometimes | occasionally | rarely/never |
| 1. How often would you ask your child to eat their food such that they could have a sweet or dessert to follow? | frequently | sometimes | occasionally | rarely/never |
| 1. How often would you help feed your child (eg, hold the spoon to put food into their mouth)? | frequently | sometimes | occasionally | rarely/never |
| 1. How often do you use food to reward your child for good behaviour? | frequently | sometimes | occasionally | rarely/never |
| 1. How often do you give food ‘treats’ to your child? | frequently | sometimes | occasionally | rarely/never |
| 1. How often do other carers (eg, grandparents) give food ‘treats’ to your child? | frequently | sometimes | occasionally | rarely/never |
| 1. How often is food allowed to be eaten in front of the TV in your home? | frequently | sometimes | occasionally | rarely/never |
| 1. How often is food served ‘buffet style’ (from the middle of the table) in your household? | frequently | sometimes | occasionally | rarely/never |
| 1. How often does your child accompany the adult to do grocery (food) shopping? | frequently | sometimes | occasionally | rarely/never |
| 1. How often does your child help you prepare food? | frequently | sometimes | occasionally | rarely/never |
| 1. How relaxing on average is the atmosphere during the evening meal with your children? | very relaxing | comfortable | tense | very stressful |
| 1. How much does your family grow its own fruits and vegetables? | very much | quite a bit | not very much | not at all |
| 1. How much do you use cooking equipment for food preparation (such as oven, microwave, food processor, electric mixer)? | very much | quite a bit | not very much | not at all |
| How much do you restrict your child’s access to the following food and beverage items   1. Fruit (fresh/canned, not dried) 2. Vegetables 3. Fruit juice 4. Dairy 5. High fat/sugar snack food 6. Carbonated drinks/Cordial 7. Water 8. Second Helpings | frequently  frequently  frequently  frequently  frequently  frequently  frequently  frequently | sometimes  sometimes  sometimes  sometimes  sometimes  sometimes  sometimes  sometimes | occasionally  occasionally  occasionally  occasionally  occasionally  occasionally  occasionally  occasionally | rarely/never  rarely/never  rarely/never  rarely/never  rarely/never  rarely/never  rarely/never  rarely/never |
| Which food groups are currently available in the home (in pantry/refrigerator/freezer)?   1. Fruit 2. Vegetables 3. Fruit juice 4. Dairy 5. Chips or similar, savoury snack biscuits, salted nuts 6. Lollies, sweets, chocolates 7. Muesli bars/breakfast bars 8. Cakes/biscuits 9. Carbonated drinks/cordial (diluted = x 5) | 0-2.9kg  0-2.9kg  0-1.9L  full fat  0-99g  0-119g  0  0-139g  0-1.9L | 3-5.9kg  3-5.9kg  2-2.9L  red. fat  100-299g  120-399g  0.5 box  140-499g  2-4.9L | 6-7.9kg  6-7.9kg  3-3.9L  red./low fat  300-699g  400-799g  1-2 boxes  500-999g  5-10.9L | ≥8kg  ≥8kg  ≥4L  low fat  ≥700g  ≥800g  >2 boxes  ≥1000g  ≥10L |
|  |  |  |  |  |
